# Supplementary material for: Resonance-enhanced three-photon luminesce via lead halide perovskite metasurfaces for optical encoding
Source: Nat Commun. 2019 May 7;10:2085. doi: 10.1038/s41467-019-10090-7 (PMC6504863; doi:10.1038/s41467-019-10090-7)
Supplement: Supplementary file 1 — Supplementary Information [file 41467_2019_10090_MOESM1_ESM.pdf]

Supplementary information for

Resonance-enhanced Three-photon Luminesce via Lead Halide Perovskite Metasurfaces for Optical Encoding

Yubin Fan[1], Yuhan Wang[1], Nan Zhang[1], Wenzhao Sun[1], Yisheng Gao[1], Cheng-Wei Qiu[2], Qinghai Song[1,3, *], Shumin Xiao[1,3, #]

[1.] State Key Laboratory on Tunable laser Technology, Ministry of Industry and Information Technology Key Lab of Micro-Nano Optoelectronic Information System, Shenzhen Graduate School, Harbin Institute of Technology, Shenzhen, China, 518055.

[2.] Department of Electrical and Computer Engineering, National University of Singapore, 4 Engineering Drive 3, Singapore 117583, Singapore.

[3.] Collaborative Innovation Center of Extreme Optics, Shanxi University, Taiyuan 030006 China.

These authors contribute equally to this research: Yubin Fan, Yuhan Wang.

Email: # shumin.xiao@hit.edu.cn; * qinghai.song@hit.edu.cn; ǂchengwei.qiu@nus.edu.sg

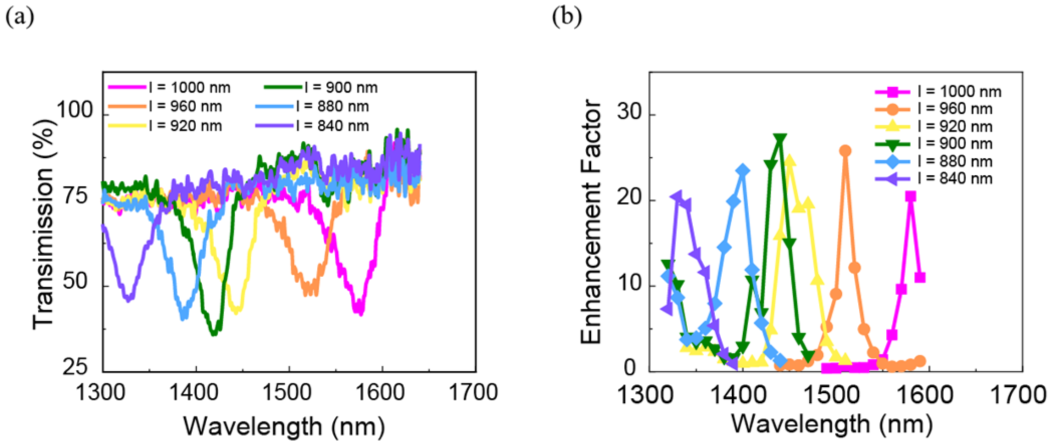

Supplementary Figure 1: The transmission and TPL enhancement factor of $MAPbBr_3$. (**a**) The transmission spectra of different perovskite metasurface with *l* = 840 nm, *l* = 880 nm, *l* = 900 nm, *l* = 920 nm, *l* = 960 nm, and *l* =1000 nm. Here the width *w* is *l*-300 nm. The insets are their corresponding high-resolution SEM images. (**b**) The enhancement factors of perovskite metasurfaces in (**a**).

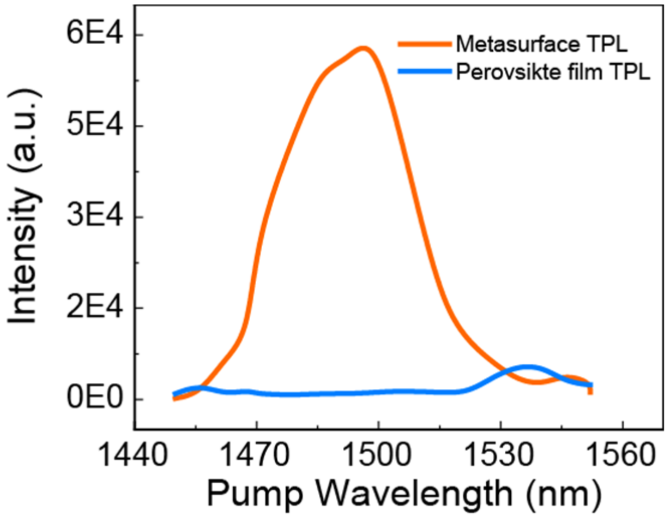

Supplementary Figure 2. The integrated photoluminescence intensity from the perovskite metasurface (orange line) and the perovskite film (blue line). Figure 4(**d**) is obtained by normalizing the emission from metasurface with the blue line.

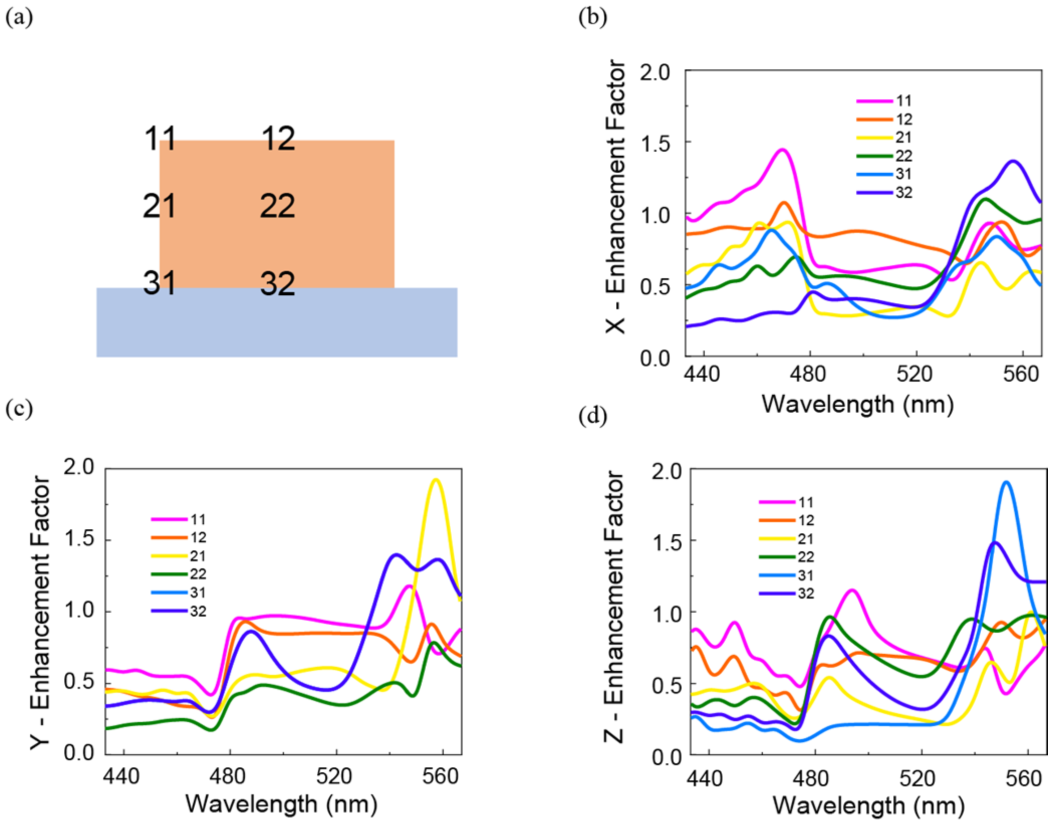

Supplementary Figure 3. The influence of the output coupling efficiency of spontaneous emission. (**a**)The schematic picture and the locations of six dipoles in perovskite metasurface. (**b**) – (**d**) The enhancement factor of the output coupling efficiency for dipoles along the x, y, and z directions, respectively.

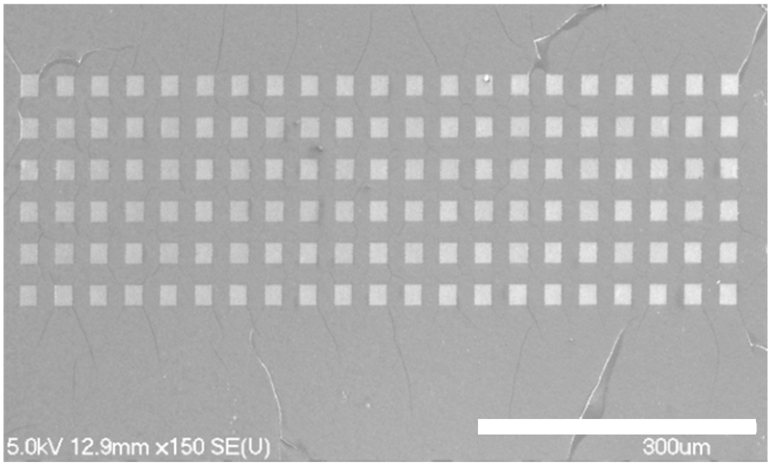

Supplementary Figure 4: The SEM image of the perovskite metasurface. Without using the guiding lines in the main text, the encoded information is invisible, scale bar is 300 μm

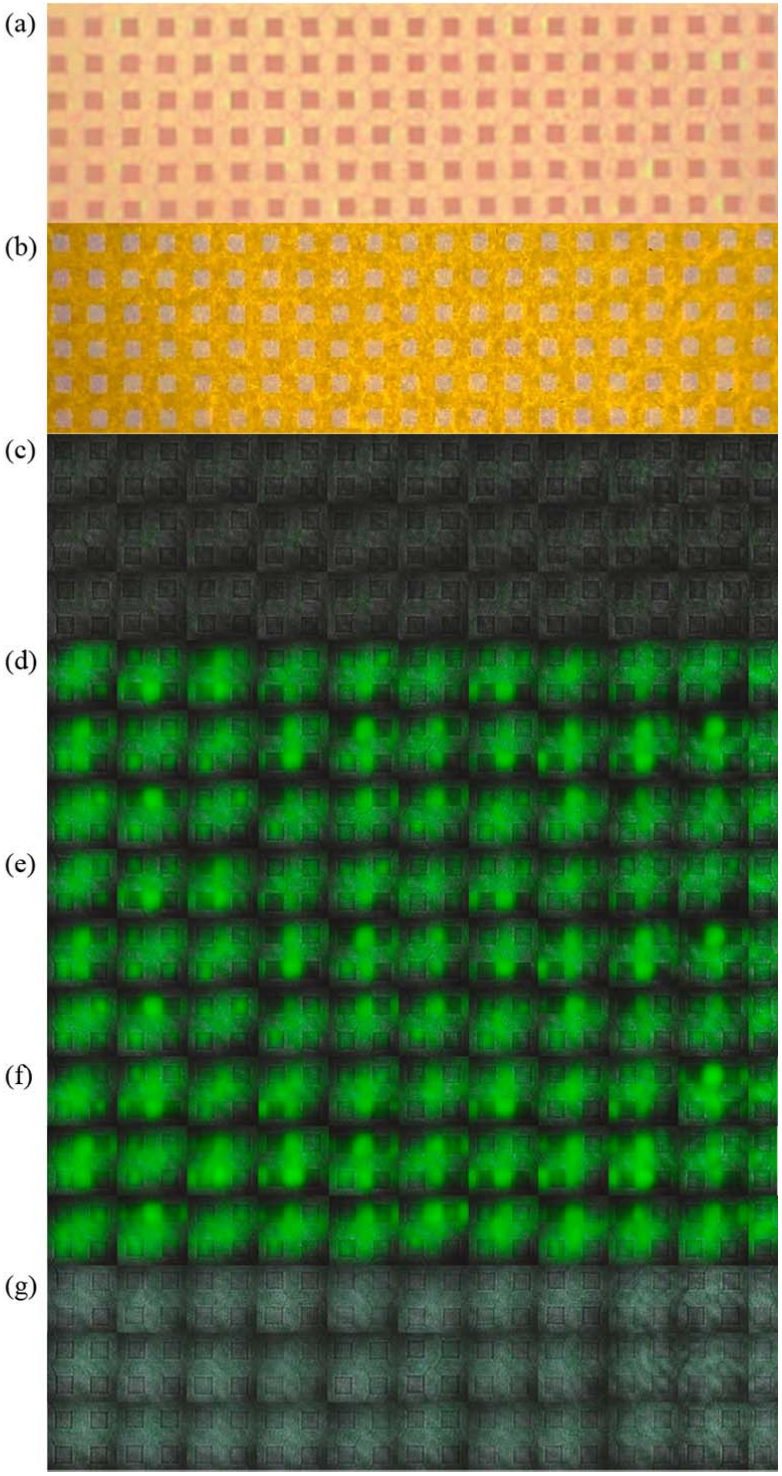

Supplementary Figure 5: The optical image under bright-field microscope, dark-field microscopy and image that metasurface pumped by non-resonant wavelengths. (**a**) optical image under brightfield microscope, (**b**) dark-field microscopy and (**c-g**) pumped by 1300 nm, 1470 nm, 1474 nm, 1480 nm and 1590 nm

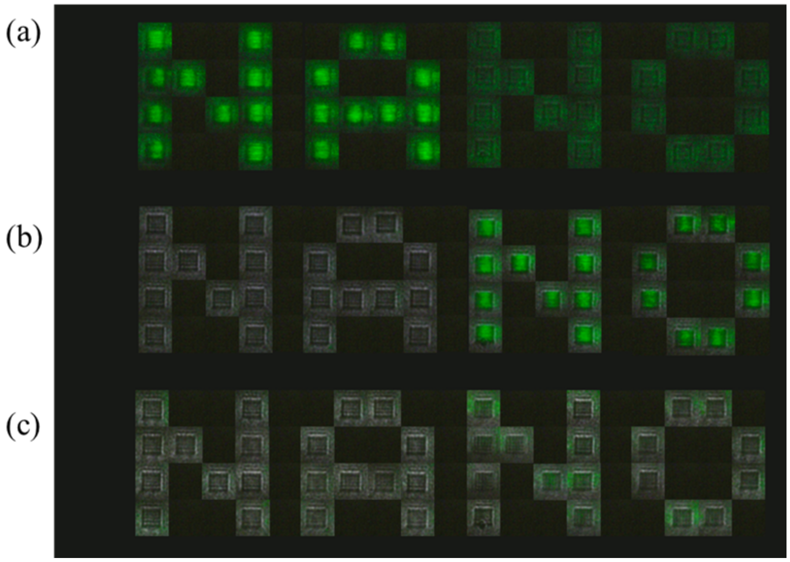

Supplementary Figure 6: The optical encryption with enhanced nonlinear process in perovskite metasurface. 'NA' is designed for working at 1500 nm and 'NO' for 1400 nm. (**a**) – (**c**) are the corresponding nonlinear photoluminescence images under different pumping wavelengths at 1500 nm, 1400 nm, and linear photoluminescence image pumped by a laser at 1350 nm. (**a**)When pumped by 1500 nm, only 'NA' is light up, (**b**) 'NO' is illuminated as for being pumped by 1400 nm.

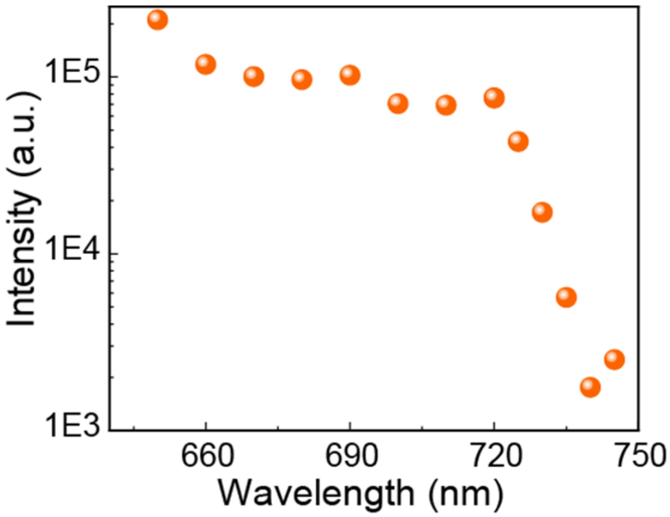

Supplementary Figure 7: **The wavelength dependent two-photon luminescence.**

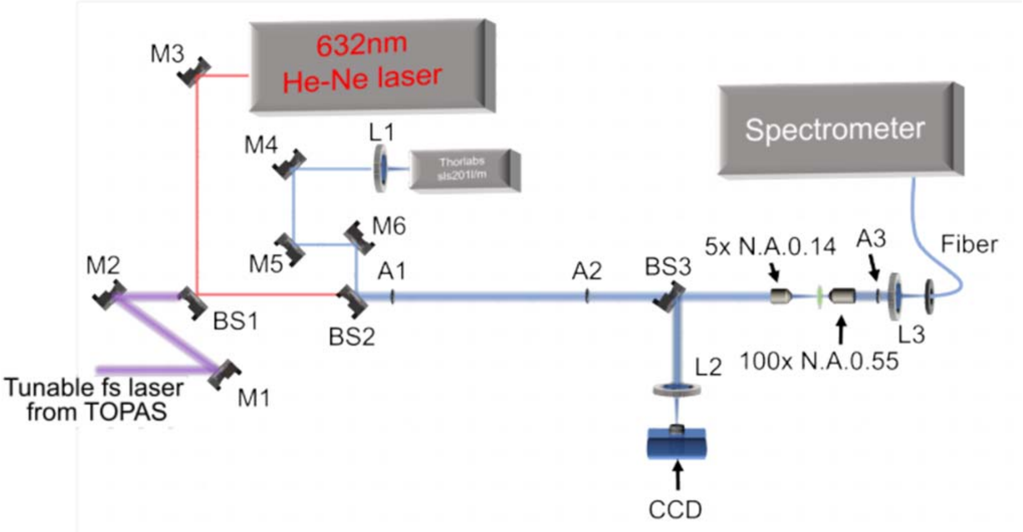

Supplementary Figure 8: The homemade microscope. M1-M6: silver mirror, BS1 – BS3: beam split, L1 - L4: lens, A1 – A3: Aperture

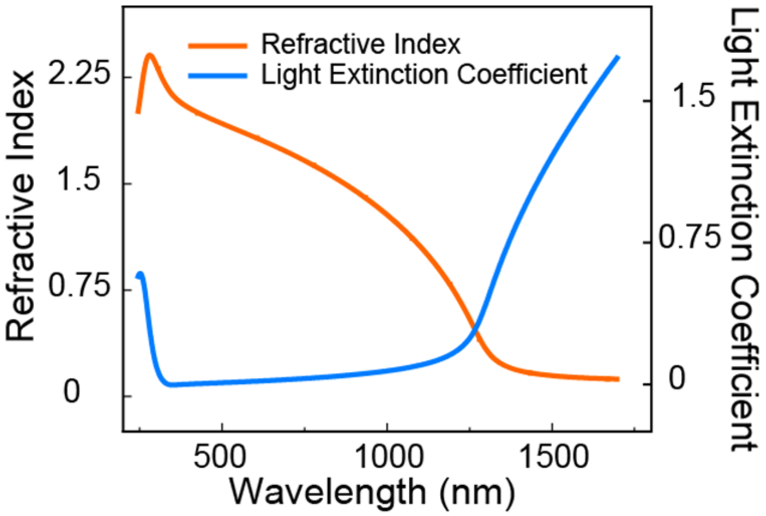

Supplementary Figure 9: Refractive index and light extinction coefficient of the ITO film in our experiment.

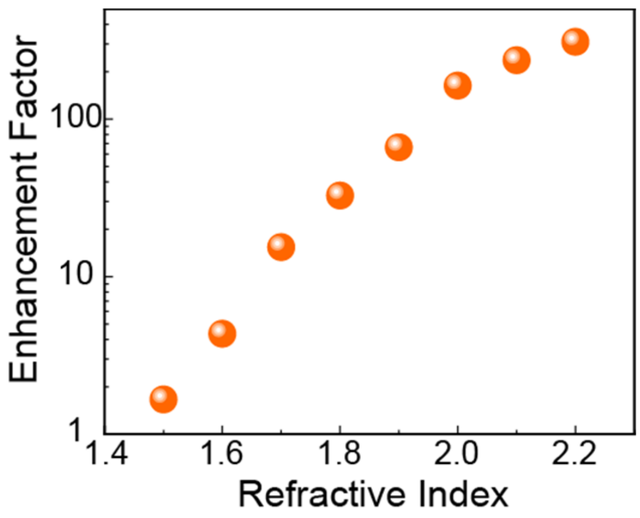

Supplementary Figure 10: The enhancement factor varies with the refractive index. The period of established simulation model is 20 with l = 960 nm and w = 660 nm.

Supplementary Note 1: The resonant enhancements in different metasurfaces

In the main manuscript, we show that the resonance of fundamental wavelength can significantly enhance the local electromagnetic field. As a result, the corresponding three-photon luminescence has been increased. Here we show that the resonant enhancement is quite generic in our perovskite metasurfaces. As shown in Supplementary Figure 1 below, the resonant peak can be shifted from 1300 nm to 1600 nm by increasing the lattice size from 840 nm to 1000 nm. Associated with the change of resonant wavelength, we can clearly see that the three-photon luminescence and the maximal enhancement factor shifts with the resonant position. We note that here the enhancement factor is obtained by comparing with the perovskite film with total area of metasurface instead of the effective area (area of the perovskite strips). In this sense, the actual enhancement factor can be much larger.

Supplementary Note 2: The normalization process for the enhancement factor, the influence of output coupling efficiency and Purcell factor on spontaneous emission

The enhancement factor in the main manuscript is normalized to the emission from unstructured perovskite films. In our manuscript, the intensities of three-photon luminescence and third-harmonic generation (THG) from both of metasurface and un-structured perovskite film with the same area are recorded. Taking the three-photon luminescence as an example, Fig. 4(d) in the main manuscript shows the experimentally obtained enhancement factor. To get these values, we measured the emissions from metasurface (orange line in Supplementary Fig. 2) and the perovskite film (blue line in Supplementary Fig. 2) at different wavelengths. The pumping density was fixed in this process. The dots in Fig. 4(d) in the main manuscript is obtained by normalizing the orange line with the blue line. The THG signals are also obtained with a similar process. As mentioned in supplementary note-1, the area of perovskite in metasurface is actually smaller than the film due to the presence of air gaps. In this sense, the actual enhancement factor can be even larger.

The out-coupling efficiency and the Purcell factors are also possible to play an essential role in the three-photon luminescence or THG. We numerically calculate the outcoupling efficiency of our metasurface with the same process as Ref. [1]. Basically, six point dipoles are embedded in the metasurfaces (see Supplementary Figure 3 (a)). The corresponding far field patterns are recorded and compared with the same dipoles in perovskite film. All the numerical calculation results are summarized in Supplementary Fig. 3 (b)-(d). It is easy to see that the enhancement factors are around 1 in all directions. This indicates that the influence of output coupling efficiency on the enhancement factor is negligibly small in our perovskite metasurface.

The Purcell factor around the photoluminescence wavelength region has also been numerically studied. As shown in Fig. 3(b) in the main manuscript, the transmission spectrum at the visible spectrum indeed has a dip around the spontaneous emission wavelength range. However, as the perovskite metasurface is designed for the near infrared wavelength, the higher order resonances are quite weak and can only be barely seen from the background (1-2% difference). As a result, its influence on the spontaneous emission cannot be as significant as Ref. [2].

Our experimental results are also consistent with the above numerical simulation. As shown in Fig. 4(b) in the main manuscript, there is no obvious additional enhancement around 525 nm even though the spectra are plotted in log scale. Therefore, we can confirm that the output coupling efficiency and the Purcell factor are not as significant as the resonant enhancement of the incident laser.

128

Supplementary Note 3: The simulation for metasurfaces with different period numbers and refractive index

The numerical simulations are performed with a finite element method software (COMSOL Multiphysics 4.3a). In the main manuscript, we have shown the simulated transmission spectra and enhancement factors of three-photon luminescence in Figure 5(b). For metasurfaces with different period numbers, we first add the number of rectangle that period we need, then we add on a 2 μm × 420 nm rectangle for each side of the rectangles as gap between Adjacent unit, finally a period port with linear polarized field vertical to grating under Floquet period boundary is added for wave excitation and perfect match layers (PML) are placed on the top and the bottom boundaries to minimize the reflection. The port range selects the boundary corresponding to the area of different period number of metasurfaces.

To simulate the enhancement factor, two frequency domains are used. One domain is used to calculate the metasurface. The other one is used to simulate perovskite film. Then only rectangles corresponding to the metasurface are collected to derive surface average of $|\mathrm{E}_{\mathrm{matesurfave}}|^6/|E_{\mathrm{film}}|^6$.

In our simulation, we directly simulated the perovskite nanostructures and got the enhancement factor. In experiment, due to the instability of the perovskite, we kept the ZEP layer on top of perovskite nanostructure to protect them. In this case, the reduced refractive index difference changes the local field enhancement and thus reduces the enhancement factor. By considering the capping layer, the enhancement factor in real experiments can be well fitted with the numerical calculations (see the dashed lines in Fig. 5(c) of the manuscript).

Supplementary Note 4: The optical imaging coding

Based on the resonant enhancement, we have designed the optical imaging encoding experiment in the main manuscript. We have shown that the encoded information is only visible when the incident laser is on resonance. We also show that the non-resonant incident laser only generates a dark or uniform green figure. Without the known polarization and wavelength, the encoded information cannot be viewed. Here we provide more experimental data to support the security of our method.

The first one is the SEM images. As the total size difference between the background and the information is only 60 nm. We find that such a tiny difference is hard to be identified in SEM image. Supplementary Figure 4 shows the top-view SEM image in Fig. 5 of the main manuscript. The encoded information ‘NANO’ cannot be identified without using the guiding lines in the main text. This makes the optical imaging encoding to be safer.

As the scattering cross sections are strongly related to the sizes of nanoparticles, some hidden information can be observed or cracked with optical microscope, especially the dark field microscope. In our experiment, we have also checked this possibility. Supplementary Fig. 5(a) and Fig. 5(b) show the bright-field and dark-field microscope images of the perovskite metasurface array. Because both the background and the information metasurfaces don’t have obvious higher-order resonances at the visible spectrum. As a result, no color difference can be observed between the ‘NANO’ and the background metasurfaces. This is different from the typical methods in literatures.

In the main manuscript, we have shown that the encoded information cannot be cracked by an incident laser at 1350 nm or linear excitation at 400 nm. Here we show that the information can be well concealed if the other laser wavelengths are used. Supplementary Fig. 5(c)-5(g) shows the corresponding images with excitation at 1300 nm, 1470 nm, 1474 nm, 1480 nm, and 1590 nm, respectively. We can see that the encoded information is invisible except the incident laser is on-resonance. Supplementary Fig. 5(d)-5(f) show that the whole image can become green if the metasurfaces are pumped harder. However, the information ‘NANO’ is still well concealed.

In additional to encoded a ‘NANO’ in a background like Fig. 5 in the main manuscript, we can also partially decorate the image to encode information. One example is shown in Supplementary Figure 6. In this figure, an image of ‘NANO’ was generated by fabricating metasurface array into a perovskite film. The ‘NANO’ can be seen with optical microscope and SEMs. However, here the encoded information is only ‘NA’. For the authorized persons, both of the polarization and wavelength are known. They can get the image ‘NA’ when they excite the metasurface with a laser at 1500 nm (see Supplementary Fig. 6(a)). Without the authorization, the random excitation can only get the dark image without information (see Supplementary Fig. 6(c)). In some cases, the hacker might scan the incident laser. They can get Supplementary Fig. 6(a) at 1500 nm and Supplementary Fig. 6(b) at 1400 nm. Without

knowing the resonant wavelength, they still don’t know which one (‘NA’ or ‘NO’) is the correct information.

Supplementary Note 5: The wavelength dependent two-photon luminescence.

The multiple exciton has the ability of enhancing the photoluminescence intensity by orders of magnitude. We experimentally verify the wavelength dependence of two-photon luminescence from our perovskite film. All the results are shown in Supplementary Fig. 7 below. With the decrease of pumping wavelength, there is a dramatic increase at around 720 nm. This is also caused by the excitonic resonance at ~ 3.4 eV [3], which can greatly enhance the multi-photon absorption process. Such kind of additional enhancement is quite interesting and worth to study in future. For our current research, as the enhancement factor is calculated by normalizing with the perovskite film, the material enhancement won’t change the final experimental results.

Since both the pure material resonance from multiple exciton resonance and the structural resonance can enhance the nonlinear processes, the combination of two effects (designing the structural resonance for the excitation of multi-exciton resonance) can further improve the nonlinear signals by orders of magnitude.

Supplementary Note 6: The set-up for optical characterizations

The linear transmittance of the metasurface was measured using a custom-built free space infrared microscope (see Supplementary Fig. 8). A compact stabilized broadband white light lamp (Thorlab SLS201L/M) is used as the light source and the metasurface is imaged using a camera by reflex observation. The incident light is weakly contracted and normally focused onto the top surface of metasurface via an objective lens (5x, N.A.0.12). The transmitted light is collected by a long working distance objective lens (Beijing PDV Instrument Co., Ltd., CCW-100x, N.A.0.55). During the experiment, we first adjust z position of the 100x objective lens to image our simple at the aperture(A3), then the image is limited by A3 to see only ~40um x 40um area of the simple. The collected signals are collected by an additional lens (50.8 mm focus length) and coupled to a spectrometer (Princeton Instrument, SpectroPro 2750i) via a multimode fiber array. Two detectors are coupled to the spectrometer, i.e. a CCD camera (Princeton Instrument, Pixis256, spectral range 190 nm-1000 nm) and a near-infrared photodetector (ID441, spectral range 900 nm – 1700 nm). The first one is applied to record the visible transmission spectrum, whereas ID441 is used to measure the near-infrared transmission spectrum.

In case of the nonlinear spectroscopic measurement, a regenerative amplified femtosecond laser (Spectra-Physics, 800 nm, repetition rate 1 kHz, pulse width 100 fs, seeded by MaiTai) is coupled to an oscillating parametric amplifier (TOPAS, wavelength range: 290 nm to 2600 nm). Then the tunable laser is guided into the homemade microspore system and focused onto the sample. The transmitted light is collected and measured by the Pixes 256 CCD camera. All of these measurements are the same as linear transmission measurement except a bandpass filter is applied to block the incident laser.

The three photon fluorescence images are collected by a 50x, N.A.0.42 long working distance objective lens, as the field of view of 100x is too narrow to collect the whole pixel. While imaging the pixel, a visible CCD simply replace the aperture(A3). A polarizer in front of the CCD to filter the THG signal.

Supplementary Note 7: The optical properties of dielectrics for numerical simulations

In order to understand and fit the experimental results, we have to accurately acquire the optical properties of all dielectric layers. The refractive index and light extinction coefficient have been measured and shown in the main text. In our experiment, there is an additional 13 nm ITO film on the glass substrate. We have also measured the optical properties of the ITO glass with optical ellipsometer. All the results are summarized in Supplementary Fig. 9 below and has been taken into accounted during the numerical calculations.

Supplementary Note 8: The simulation for enhancement factor varies with the refractive index.

The resonance of dielectric metasurface and enhancement factor depend heavily on the refractive index of the composition material. As shown in Supplementary Fig. 10 below, for metasurface with infinite periodicity with the increase of the refractive index, the enhancement factor grows almost exponentially. For most of dyes doped polymers which is normally used as nonlinear optical media, the enhancement factor can be negligible due to the low refractive index (n ~ 1.5-1.6). However, the lead halide perovskite can form strong resonance benefit from the higher refractive index (n ~ 1.8-2.5). As a result, much higher local field enhancement can be obtained with enhancement factor more than 100 times. As a conclusion, utilizing perovskite metasurface to enhance the resonance of fundamental beam is quite feasible.

Supplementary References:

1. Lin, K. et al., Perovskite light-emitting diodes with external quantum efficiency exceeding 20 per cent. *Nature* **562**, 245 (2018).
2. Tiguntseva, E. Y. et al., Light-emitting halide perovskite nanoantennas. *Nano Lett.* **18**, 1185-1190 (2018).
3. Leguy, A. M. et al., Experimental and theoretical optical properties of methylammonium lead halide perovskites. *Nanoscale* **8**, 6317-6327(2016)
